# Epigonal Conditioned Media from Bonnethead Shark, *Sphyrna tiburo*, Induces Apoptosis in a T-Cell Leukemia Cell Line, Jurkat E6-1

**DOI:** 10.3390/md11093224

**Published:** 2013-08-26

**Authors:** Catherine J. Walsh, Carl A. Luer, Jennifer E. Yordy, Theresa Cantu, Jodi Miedema, Stephanie R. Leggett, Brittany Leigh, Philip Adams, Marissa Ciesla, Courtney Bennett, Ashby B. Bodine

**Affiliations:** 1 Marine Immunology Program, Mote Marine Laboratory, 1600 Ken Thompson Parkway, Sarasota, FL 34236, USA; 2 Marine Biomedical Program, Mote Marine Laboratory, 1600 Ken Thompson Parkway, Sarasota, FL 34236, USA; E-Mails: caluer@mote.org (C.A.L.); jyordy@mote.org (J.E.Y.); theresacantu23@gmail.com (T.C.); jodi.miedema@yahoo.com (J.M.); scuba.steph@hotmail.com (S.R.L.); bleigh@mail.usf.edu (B.L.); philip.adams1215@gmail.com (P.A.); mciesla@mail.usf.edu (M.C.); cbennett@mote.org (C.B.); 3 Department of Animal and Veterinary Sciences, Clemson University, Clemson, SC 29634, USA; E-Mail: abodine@clemson.edu

**Keywords:** epigonal organ, bonnethead shark, Jurkat, apoptosis, tumor cell line

## Abstract

Representatives of Subclass Elasmobranchii are cartilaginous fish whose members include sharks, skates, and rays. Because of their unique phylogenetic position of being the most primitive group of vertebrates to possess all the components necessary for an adaptive immune system, the immune regulatory compounds they possess may represent the earliest evolutionary forms of novel compounds with the potential for innovative therapeutic applications. Conditioned medium, generated from short term culture of cells from the epigonal organ of bonnethead sharks (*Sphyrna tiburo*), has been shown to have potent reproducible cytotoxic activity against a variety of human tumor cell lines *in vitro*. Existing data suggest that epigonal conditioned medium (ECM) exerts this cytotoxic activity through induction of apoptosis in target cells. This manuscript describes apoptosis induction in a representative tumor cell line, Jurkat E6-1, in response to treatment with ECM at concentrations of 1 and 2 mg/mL. Data indicate that ECM exposure initiates the mitochondrial pathway of apoptosis through activation of caspase enzymes. Future purification of ECM components may result in the isolation of an immune-regulatory compound with potential therapeutic benefit for treatment of human cancer.

## 1. Introduction

While a few pharmaceuticals have originated from the marine environment or been derived from tissues of marine inhabitants, marine organisms represent a relatively untapped source of potentially novel compounds. In particular, elasmobranch fishes (cartilaginous fish whose members include the sharks, skates, and rays) may provide a unique and underutilized source of potential therapeutic agents. With anecdotal observations that sharks, skates, and rays have a low incidence of disease in general, but specifically a low incidence of documented malignant tumors, understanding the role of the elasmobranch immune system in this apparent resistance would open the door for new areas of research.

Although research in this area may potentially translate into applications for human health, a basic understanding of the elasmobranch immune system components and how they function is essential. Immunologically, elasmobranch fishes fill a unique niche in that they are phylogenetically the most primitive group of vertebrates to possess all the components necessary for an adaptive immune system [1,2,3,4]. As in higher vertebrates, elasmobranchs possess thymus and spleen, but in the absence of bone marrow and lymph nodes, these fish have evolved unique lymphomyeloid tissues, namely the epigonal and Leydig organs. Because they are specific to elasmobranchs, research in our lab has focused on the epigonal and Leydig organs, with efforts focused on *in vitro* culture of cells collected from the epigonal organ. Optimization of short-term culture of elasmobranch immune cells has facilitated investigation of cytokine-like factors derived from conditioned culture medium from elasmobranch epigonal cells (epigonal conditioned medium, ECM). Specifically, media conditioned by cultures of epigonal tissue from bonnethead sharks (*Sphyrna tiburo*) contains potent growth inhibitory activity against a number of tumor cell lines [5], while only limited growth inhibitory activity against normal cells [6]. Among the cell lines tested, the Jurkat E6-1 cell line, derived from an acute T-cell leukemia [7], was shown to be among the most sensitive to growth inhibition effects resulting from exposure to ECM. Previous reports indicate that cytotoxic activity of ECM against mammalian tumor cell lines proceeded through mechanisms of apoptosis [5,6]. In this paper, pathways of apoptosis initiated by exposure to ECM in Jurkat cells are described.

**Figure 1:**
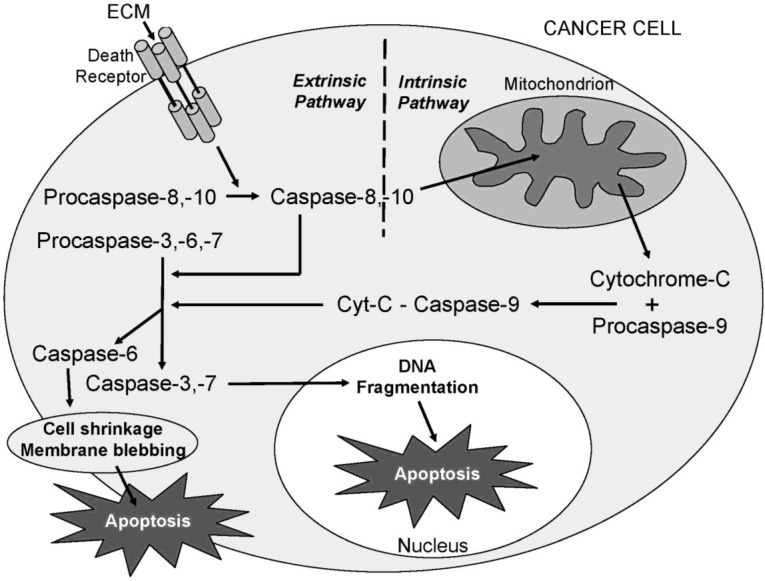
Schematic diagram showing the activation of selected initiator and effector caspase pathways resulting in apoptosis.

Caspases (cysteine-dependent aspartate-directed proteases) belong to a highly conserved family of cysteine proteases with specificity for aspartic acid residues on their substrates. Caspase enzymes play a central role in apoptosis and are classified as initiators or effectors (executioners), depending on point of entry into the apoptotic cascade. To date, ten major caspases have been identified and broadly categorized as initiators (caspases-2, -8, -9, -10), effectors/executioners (caspases-3, -6, -7), or inflammatory caspases (caspases-1, -4, -5) (reviewed in [8]). Initiator caspases are the first to be activated, and serve as the initial steps in a cascade effect that activates downstream effector or executioner caspases. Initiator caspases (-8, -9, and -10) are closely coupled to pro-apoptotic signals and once activated, the execution phase of apoptosis is triggered and downstream effector caspases (-3, -6, and -7) are cleaved and activated, which, in turn, cleave cytoskeletal and nuclear proteins, such as PARP, and ultimately result in cell apoptosis. A schematic diagram of selected initiator and effector caspase activation pathways resulting in apoptosis is shown in Figure 1.

## 2. Results and Discussion

### 2.1. Growth Inhibition Assay

**Figure 2:**
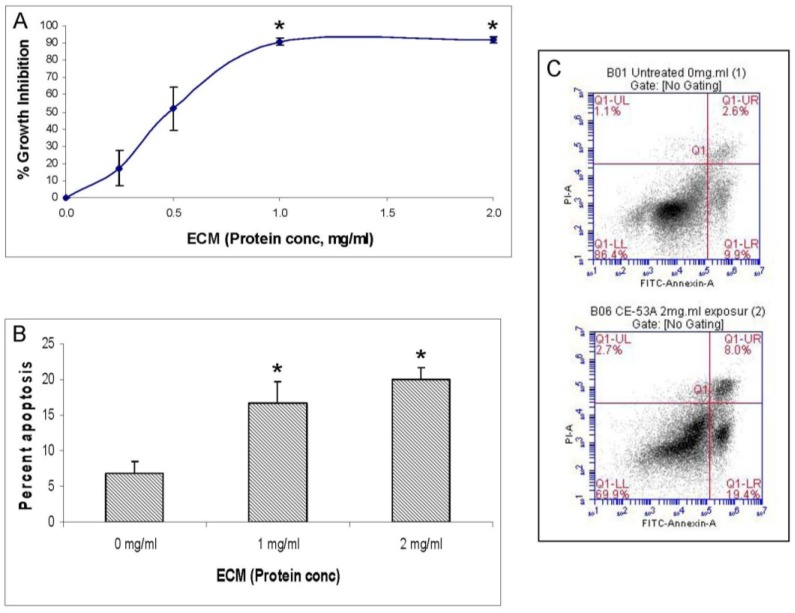
Effects of epigonal conditioned medium (ECM) on cell growth and apoptosis in Jurkat cells. (**a**) Growth inhibition of Jurkat cells (*N* = 11) treated with ECM for 72 h measured using MTT. * Significantly greater (*P* < 0.05) than control; (**b**) Apoptosis in Jurkat cells treated with ECM for 24 h (*N* = 3), measured using flow cytometric detection of annexin V binding. * Significantly greater than untreated control (*P* = 0.0057 and 0.0197, respectively); (**c**) Histogram from ECM treated Jurkat cells showing binding of annexin V in flow cytometry; untreated control (top), 2 mg/mL ECM (bottom).

Growth inhibitory activity of ECM was assessed using a T-cell leukemia cell line, Jurkat E6-1 (ATCC TIB 152). Growth inhibition was quantified using the MTT assay [9], an assay that measures the ability of live cells to convert the tetrazolium salt MTT (3-(4,5-dimethylthiazol-2-yl)-2,5-diphenyl tetrazolium bromide) to a formazan product via mitochondrial enzymes. At ECM concentrations of 1 and 2 mg/mL, inhibition of Jurkat cell growth after 72 h was greater than 90% (Figure 2A). At 0.5 mg/mL, growth inhibition was greater than 50%. Growth inhibition in response to 1 and 2 mg/mL ECM was significantly (*P* < 0.05) greater than control. Reduction in conversion of MTT to the formazan product by ECM-treated Jurkat cells indicates that ECM induces dose-dependent inhibition of Jurkat cell growth.

### 2.2. Annexin V Assay

Annexin V is a phospholipid binding protein with high affinity for phosphatidylserine. In viable cells, phosphatidylserine is located on the cytoplasmic surface of cell membranes. In cells undergoing early cell membrane changes associated with apoptosis, phosphatidylserine is translocated from the inner membrane to the outer part of the plasma membrane where it can be detected using fluorescently labeled annexin V conjugates [10]. After 24 h, 16.72% ± 2.97% (SEM) of the Jurkat cells treated with 1 mg/mL ECM and 19.97% ± 1.76% (SEM) of the cells treated with 2 mg/mL ECM were shown to bind annexin V (Figure 2B). Binding of annexin V was significantly greater in Jurkat cells treated with 1 mg/mL ECM (*P* = 0.0057) and 2 mg/mL ECM (*P* = 0.0197) compared to untreated control cells. A flow cytometry histogram indicating a shift in the percentage of cells binding annexin V in response to ECM treatment is shown in Figure 2C. These results indicate that ECM-treated Jurkat cells undergo apoptosis and that apoptotic processes are likely involved in the observed growth inhibition. In this histogram, an insignificant (*P* = 0.202) increase in the number of dead cells (Q1-UR; Figure 2C) was also observed with ECM treatment.

### 2.3. Caspase Activity Assays

**Figure 3:**
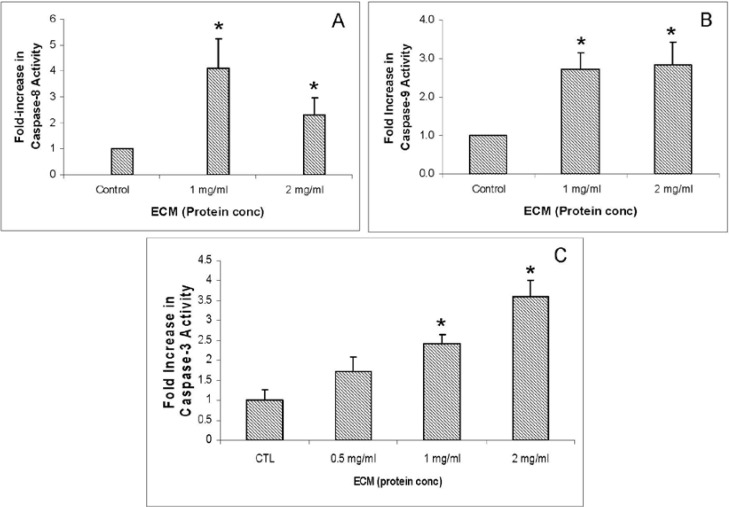
Activation of caspase enzymes in Jurkat cells by ECM treatment. (**a**) Caspase-8; (**b**) Caspase-9; (**c**) Caspase-3. Activity of caspases-8 and -9 were measured using a colorimetric microplate assay; activity of caspase-3 was measured using a fluorimetric microplate assay. * Significantly different (*P* < 0.05) from control.

To determine whether ECM exposure activates the caspase cascade in Jurkat cells, the functional activity of two initiator caspases (-8, -9) and one effector caspase (-3) was assessed in cell lysates from untreated and ECM-treated Jurkat cells. Results from enzyme activity assays are shown in Figure 3, and indicate increased caspase activity with exposure to ECM. Exposure to ECM resulted in a greater than 4-fold (4.11 ± 1.13, *N* = 4, *P* < 0.05) increase in the activity of the initiator caspase-8 (Figure 3A) in response to 1 mg/mL ECM and a greater than 2-fold (2.31 ± 0.67, *N* = 4, *P* < 0.05) increase in activity in response to 2 mg/mL ECM compared to untreated cells. Activity of the initiator caspase-9 (Figure 3B) was also significantly increased compared to untreated cells. At 1 mg/mL ECM, caspase-9 activity was 2.73 ± 0.41 (SEM)-fold and at 2 mg/mL ECM, caspase-9 activity was 2.83 ± 0.59 (SEM)-fold greater than in untreated Jurkat cells. Activity of the terminal caspase, caspase-3, was also increased in Jurkat cells treated with ECM compared to untreated controls (Figure 3C). The highest caspase-3 activity was observed in response to 2 mg/mL ECM, with a 4.8 ± 0.16-fold (*N* = 3, *P* < 0.05) increase in relative fluorescence compared with untreated controls. In response to 1 mg/mL ECM, the fold increase was 4.3 ± 0.22 (*P* < 0.05, *N* = 3). These results indicate significant activation of key enzymes in the caspase cascade—caspases-8, -9, and -3—in response to exposure to ECM for 24 h. All apoptotic pathways end in activation of caspase-3, so observation of caspase-3 activation only provides additional evidence that apoptosis is occurring in target cells. Activation of caspase-8 is generally triggered through signals transmitted by death receptors (TRAIL or Fas) on the cell surface, but apoptosis can proceed either through the mitochondria (intrinsic) or bypass the mitochondria (extrinsic). Activation of caspase-9, however, can only occur in response to mediators released from the mitochondria, and thus indicates involvement of the mitochondria in apoptosis. Thus, observations reported here of significant activation of caspases-8, -9, and -3 are consistent with progression of apoptosis through the mitochondrial pathway.

### 2.4. Western Blotting

In addition to functional activity assays, expression of specific apoptotic pathway intermediates at the protein level was assessed in ECM-treated Jurkat cells through Western blotting with monoclonal antibodies against caspases-3, -6, -7, -8, -9, and -10, cleaved caspases -3, -7, -8, and -9, apoptotic pathway promoters Smac/DIABLO, APAF-1, FADD, and apoptotic pathway inhibitors FLIP, PARP, and XIAP.

**Figure 4:**
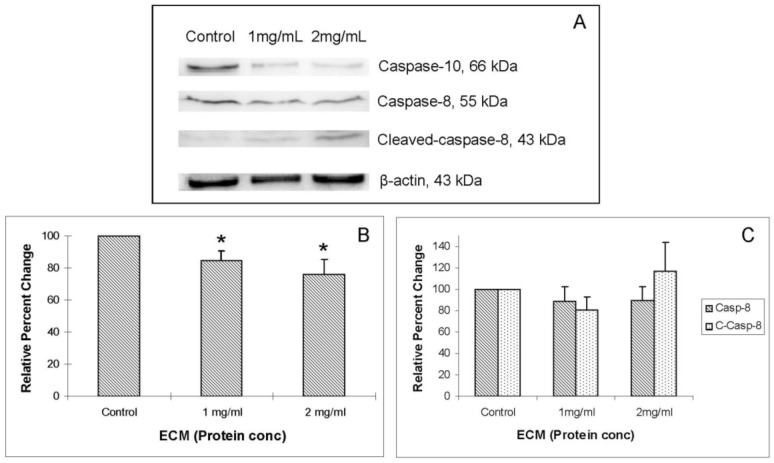
Western blot analysis demonstrating relative concentrations of caspase-10, caspase-8, and cleaved caspase-8 in Jurkat cells treated with ECM for 24 h, using β-actin as protein loading control. (**a**) Western blot images and relative band densities for (**b**) caspase-10; and (**c**) caspase-8 and cleaved caspase-8. * Significantly different (*P* < 0.05) from control.

**Figure 5:**
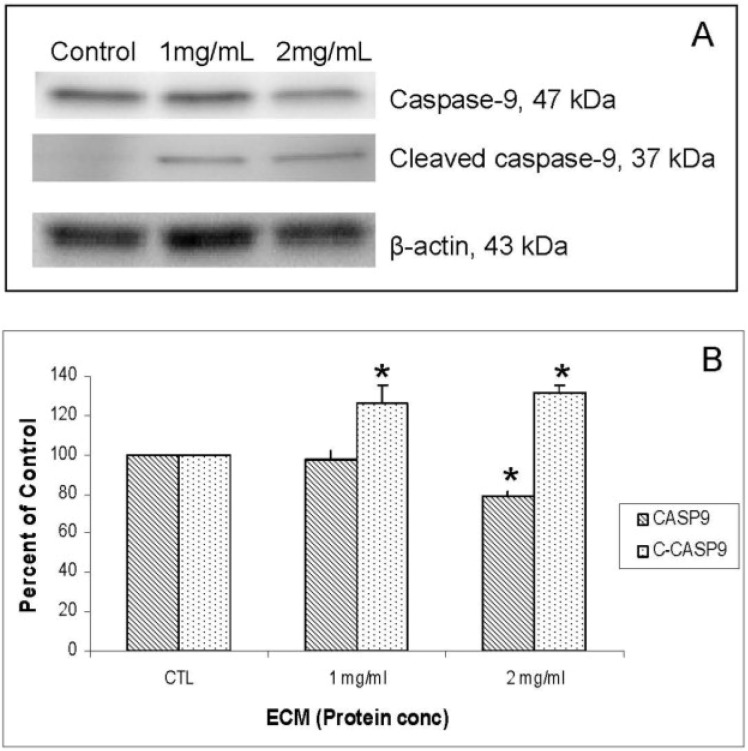
Western blot analysis demonstrating relative concentrations of caspase-9 and cleaved caspase-9 in Jurkat cells treated with ECM for 24 h, using β-actin as protein loading control. (**a**) Western blot images; (**b**) relative band densities for caspase-9 and cleaved caspase-9. * Significantly different (*P* < 0.05) from control.

Western blot images demonstrating relative concentrations of initiator caspases -8 and -10, as well as cleaved caspase-8, are shown in Figure 4A. Figure 4B,C show relative band densities, normalized to β-actin as protein loading control, of each of these molecules in ECM-treated Jurkat cell lysates compared with control. A significant (*P* < 0.05) decrease in the amount of full-length caspase-10 was observed after treatment with both 1 and 2 mg/mL ECM, which demonstrates activation of pro-caspase-10 to cleaved caspase-10. Differences in full-length and cleaved caspase-8 expression were not significant, although visually there appeared to be a difference in expression of these proteins in ECM-treated cells. These images demonstrate significant activation of caspase-10. A significant effect on protein expression of caspase-8 is not strongly observed through Western blotting. Once caspases-8 and -10 are activated, caspase-9 is cleaved, followed by activation of effector caspases-6 and -7, and finally by the terminal caspase, caspase-3. Western blot images demonstrating relative concentrations of caspase-9 and cleaved caspase-9 in Jurkat cells treated with ECM for 24 h are shown in Figure 5A. Relative band densities, using β-actin as protein loading control, are shown in Figure 5B. Full-length caspase-9 was significantly reduced in response to 2 mg ECM/mL (78.58% ± 2.36% of control; *P* = 0.015; one-way ANOVA). Production of cleaved caspase-9 is apparent, with an increase in band density over untreated cells observed following 1 mg/mL ECM treatment (125.89% ± 9.74%, *N* = 4, *P* = 0.029, *t*-test) and 2 mg/mL ECM treatment (131.20% ± 4.34%, *P* = 0.029, *t*-test). Activation of caspase-9 indicates progression of apoptosis through the mitochondrial (intrinsic) pathway.

**Figure 6:**
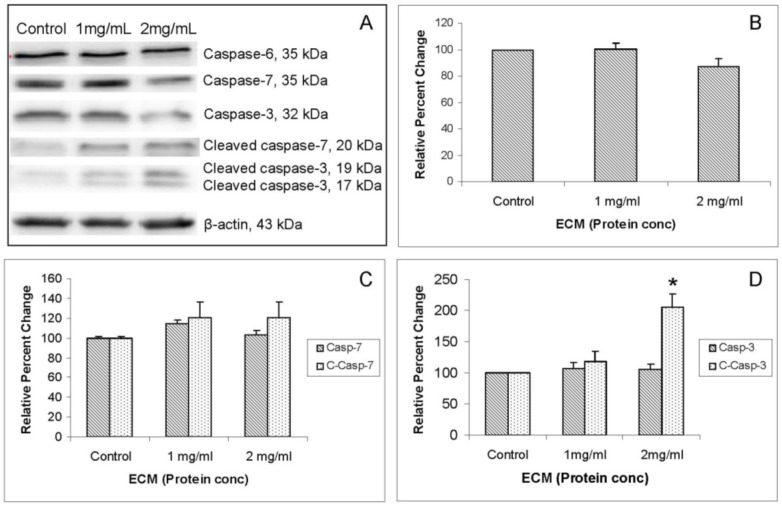
Western blot analysis demonstrating relative concentrations of caspases -6, -7, and -3 and cleaved caspase -7 and -3 in Jurkat cells treated with ECM for 24 h, using β-actin as protein loading control. (**a**) Western blot images; and relative band densities for (**b**) caspase-6; (**c**) caspase-7 and cleaved caspase-7; and (**d**) caspase-3 and cleaved caspase-3. * Significantly different from untreated control.

Western blot images demonstrating relative concentrations of caspase-6, -7, and -3 and cleaved caspases -7 and -3 in Jurkat cells treated with ECM for 24 h are shown in Figure 6A. Corresponding relative band densities are shown in Figure 6B,C. Caspase-6 is one of the major executioner caspases functioning in cellular apoptotic processes [11]. With full-length caspase-6 (35 kDa), there was very little detectable change in protein expression with ECM treatment (Figure 6A), although there was a slight (86.89% ± 6.52% of control), but statistically insignificant, decrease in expression at 2 mg/mL (Figure 6B). No significant differences in expression of full length caspase-6 were detected. Likewise, with caspase-7, there were no detectable changes in expression of full-length caspase-7 following ECM treatment for 24 h (Figure 6A,C). A slight increase (120% ± 15%) was seen in amount of cleaved caspase-7 in response to treatment with 2 mg/mL ECM, but this expression did not differ significantly from control. With caspase-3, there were slight decreases in amount of protein for full-length caspase protein (Figure 6A,D). Amounts of cleaved caspase-3 were increased by 117.72% ± 17.07% in response to 1 mg/mL and 205.84% ± 20.64% in response to 2 mg/mL (*P* = 0.037; one way ANOVA; Figure 6D). These observations indicate cleavage, and consequently, activation of initiator caspase-9 and terminal caspase-3. No significant differences in expression of proteins for caspase-6 or -7 were observed. Molecular pathways in Jurkat cells treated with ECM at concentrations of 1 and 2 mg protein/mL for 24 h indicate activation of apoptotic pathways and progression of cell death through apoptotic mechanisms.

**Figure 7:**
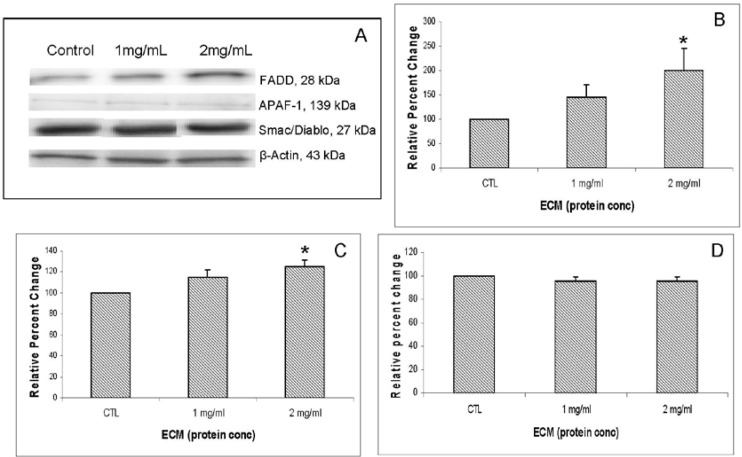
Western blot analysis demonstrating effects of 24 h ECM treatment on expression of apoptotic pathway proteins FADD, APAF-1, and Smac/DIABLO. Western blot images (**a**) and relative band densities for (**b**) FADD; (**c**) APAF-1; and (**d**) Smac/DIABLO, using β-actin as protein loading control. *Significantly different (*P* < 0.05) from untreated control.

**Figure 8:**
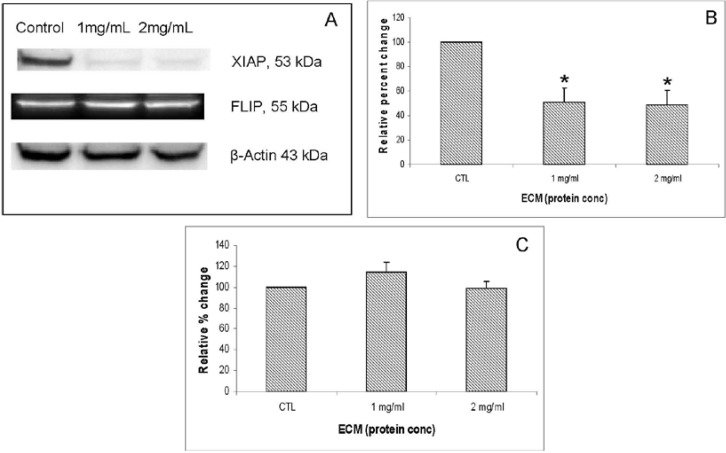
Western blot analysis demonstrating effects of 24 h ECM treatment on expression of apoptotic pathway proteins XIAP and FLIP. (**a**) Western blot images; and relative band densities for (**b**) XIAP; and (**c**) FLIP, using β-actin as protein loading control. * Significantly different (*P* < 0.05) from untreated control.

Other apoptosis pathway intermediates that were examined for their role in ECM-induced apoptosis in Jurkat cells include regulators involved in activation (FADD, APAF-1, Smac/DIABLO) as well as inhibitors of apoptosis (FLIP, XIAP, PARP). Western blotting results for FADD, APAF-1, and Smac/DIABLO are shown in Figure 7. Western blotting results for inhibitors FLIP and XIAP are shown in Figure 8. FADD (Fas-associated via death domain) functions as an apoptotic adaptor molecule that recruits caspase-8 or -10 to activated Fas (CD95) or TNFR-1 to form a death-inducing signaling complex (DISC) [12]. Relative band densities demonstrating effects of 24 h ECM on expression of FADD through Western blotting are shown in Figure 7B. With FADD, relative band density changed in response to 1 mg/mL (144.73% ± 25.45%, *N* = 3) and in response to 2 mg/mL (199.32% ± 45.2%, *N* = 3, *P* <0.05, one-way ANOVA followed by Tukey’s). APAF-1 (apoptotic protease activating factor 1) is a cytoplasmic protein involved in apoptosis by forming part of the apoptosome that binds and activates caspase-9. Relative band densities for APAF-1 are shown in Figure 7C. APAF-1 changed in relative band density by 114.79% ± 6.82% at 1 mg/mL ECM and 124.98% ± 6.07% at 2 mg/mL ECM (*P* = 0.021, one way ANOVA followed by Tukey’s). Smac/DIABLO is a mitochondrial protein that functions as an apoptotic promoter by translocating to the cytosol and activating caspases in the cytochrome c/APAF-1/caspase-9 pathway and opposing inhibitory activity of inhibitor of apoptosis proteins (IAP), including XIAP [13]. No detectable change in expression of Smac/DIABLO was observed among ECM treatments. In summary, results indicate an increase in FADD and APAF-1 expression, but no change in expression of Smac/DIABLO. An increase in FADD expression indicates that ECM is involved in recruitment of FADD to the receptor, possibly through binding to TRAIL receptor [14], which would also be consistent with observed increased in caspase-8 activity. The increase in APAF-1 expression provides evidence that ECM initiates pathways that involve apoptosome formation and activation of caspase-9 [15]. Logically, a translocation of Smac/DIABLO would be involved in this process as well, but a significant change in expression of this molecule was not observed. This is not unexpected; however, since the whole cell lysate method used in these experiments does not provide information about protein localization within the cell.

XIAP (X-linked inhibitor of apoptosis) encodes a potent apoptotic suppressor protein that functions by suppressing activities of caspases -3 and 7 [13]. FLIP (FLICE-like inhibitory protein), also known as CFLAR (CASP8 and FADD-like apoptosis regulator), is structurally similar to caspase-8 and regulates apoptosis by functioning as a crucial link between cell survival and cell death pathways by inhibiting TNFRSF6-mediated apoptosis [16,17]. As the major protein that prevents caspase-8 from activation by death receptors, c-FLIP protein can be recruited to the death-inducing signaling complex (DISC) to inhibit caspase-8 activation [18,19]. Western blot images demonstrating effects of 24 h ECM treatment on expression of apoptotic pathway inhibitor proteins, XIAP and FLIP, are shown in Figure 8A. Graphs representing relative band densities are shown in Figure 8B,C. XIAP was strongly affected by ECM treatment, with a relative band density 51.13% ± 11.29% of control at 1 mg/mL and 48.24% ± 12.14% of control at 2 mg/mL. These differences were significant at 1 mg/mL (*P* = 0.014, One way ANOVA Tukey’s) and 2 mg/mL (*P* = 0.01, Tukey’s). These results indicate a strong down-regulation of the key inhibitor protein, XIAP. Down-regulation of XIAP functions in facilitating progression of apoptotic pathways towards cell death by inhibiting caspase activity. Relative band density of FLIP did not change significantly in response to ECM treatment at either 1 or 2 mg/mL. Since FLIP is involved in inhibiting recruitment of caspase-8 [17], the absence of significant effects on expression of this inhibitor protein indicates no inhibition of apoptosis proceeding through caspase-8 recruitment and activation.

**Figure 9:**
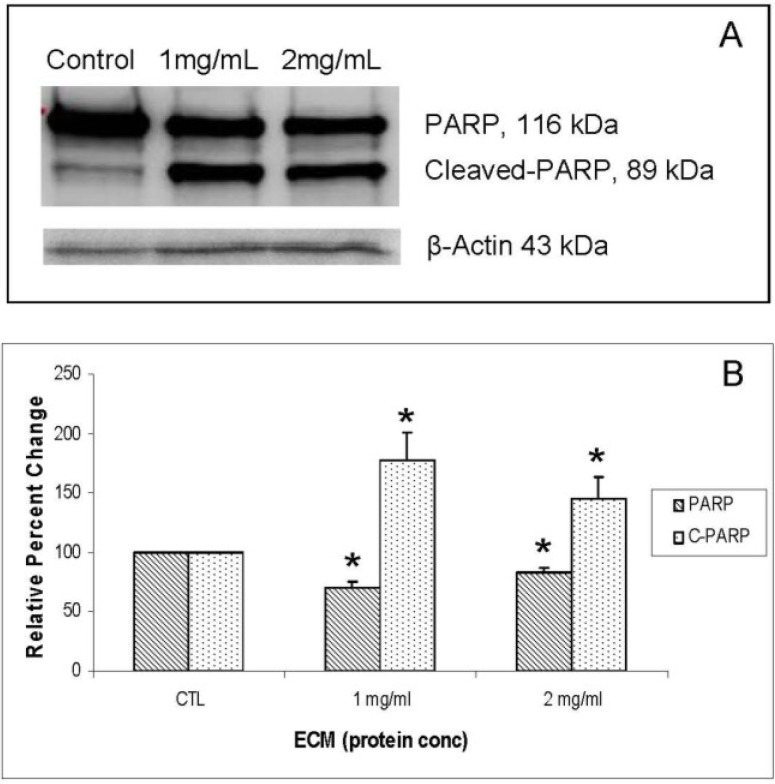
Western blot analysis demonstrating effects of 24 h ECM treatment on expression of apoptotic pathway protein PARP. (**a**) Western blot images; (**b**) relative band densities for PARP and cleaved PARP, using β-actin as protein loading control. * Significantly different from untreated control.

Another protein involved in inhibition of apoptosis is PARP (poly ADP-ribose polymerase), a nuclear protein that plays an important role in DNA repair [20] and is inactivated by enzymatic cleavage. Once the enzyme is inactivated by caspase-3, DNA repair is prevented, which allows DNA fragmentation, and thus apoptosis, to proceed. Effects of ECM treatment on the apoptotic inhibitor, PARP, were investigated using Western blotting, with results shown in Figure 9A (blot) and 9B (relative band densities for PARP/cleaved PARP). Cleavage of PARP was strongly observed in ECM-treated Jurkat cells, as evidenced by significant (*P* < 0.05) decreases in full-length PARP and significant increases in cleaved PARP at both 1 and 2 mg ECM protein/mL. These results indicate that the nuclear DNA repair protein, PARP, is inactivated in Jurkat cells treated with ECM.

### 2.5. Antibody Array

**Figure 10:**
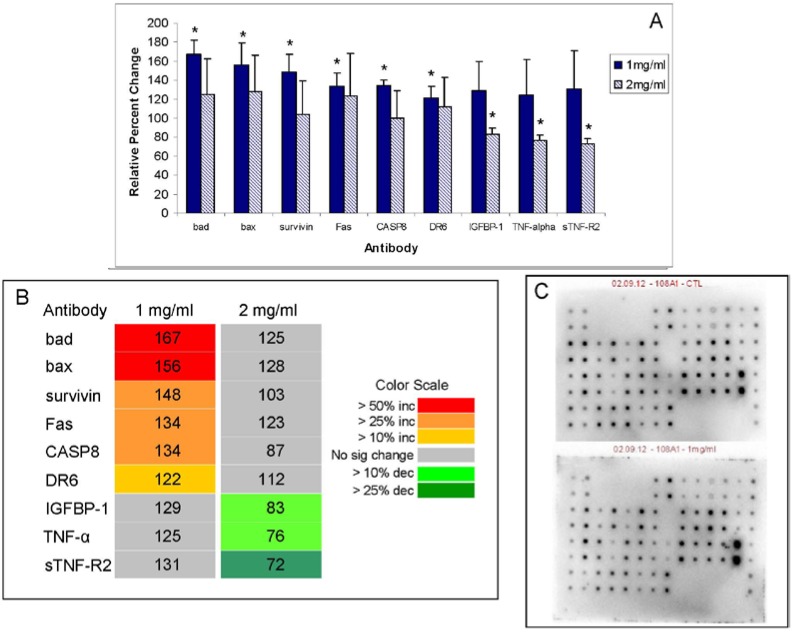
Relative percent change in expression of apoptotic pathway proteins in Jurkat cell lysates following 24 h treatment with ECM. Protein expression was determined using an antibody array (RayBio) containing 43 different antibodies for apoptotic pathway intermediates. Results are expressed as relative percent change compared to control (untreated) Jurkat cells using a heat map according to the color scale on the lower right. Only proteins that were significantly different (*P* < 0.05) compared to control at either 1 or 2 mg/mL ECM are shown. (**a**) Relative expression of intermediates significantly increased or decreased (d) from control at either 1 or 2 mg/mL. * Significantly different from control (*P* < 0.05); (**b**) heat map showing relative change in expression of proteins with significant increases or decreases; (**c**) representative array blot. *N* = 4 trials for the experiment.

An antibody array that measures 43 different apoptotic pathway intermediates was utilized to assess effects of ECM on Jurkat cells. The change in expression of apoptotic pathway proteins in Jurkat cell lysates detected on the antibody array following 24 h treatment with ECM is shown in Figure 10. Results are expressed as relative percent change compared to control (untreated) Jurkat cells using a graph representing relative band densities (Figure 10A) and a heat map (Figure 10B). A representative image of antibody arrays from control and ECM-treated (1 mg/mL) Jurkat cell lysates is also given (Figure 10C). Responses to ECM varied with concentration; of the 43 proteins that were on the array, only 9 were significantly (*P* < 0.05) changed from control in response to treatment with either 1 or 2 mg/mL ECM (ANOVA, *P* < 0.05). No pathway intermediates changed significantly in response to both ECM concentrations. The nine proteins which changed significantly at either 1 or 2 mg/mL ECM included the mitochondrial proteins BAD and BAX, apoptosis inhibitor, survivin, initiator caspase-8, death receptors DR6 and Fas, insulin growth factor binding protein IGFBP-1, and TNF family members TNF-α and sTNF-R2.

BAD and BAX are mitochondrial proteins that belong to the bcl-2 family of apoptosis regulators [21]. BAD, also known as BCL2-associated agonist of cell death, positively regulates apoptosis by forming heterodimers with BCL-XL and BCL-2 and reversing their repressor activity. BAX, also known as BCL2-associated X protein, activates apoptosis by opening mitochondrial voltage-dependent anion channel (VDAC) which leads to loss in membrane potential and subsequent release of cytochrome c from the mitochondria. BAX and BAD were both up-regulated greater than >150% in response to 1 mg/mL ECM (*P* < 0.05), but not significantly up-regulated in response to 2 mg/mL. Although an antibody for cytochrome c was included on the array, expression of cytochrome c was not significantly changed in response to ECM treatment. An increase in cytochrome c release, however, was previously demonstrated using mitochondrial and cytosolic fractions from another cell line, a B-cell lymphoma (Daudi) exposed to ECM [5,6]. Similar to Smac/DIABLO, it is likely that no change was observed in these experiments with ECM-treated Jurkat cells because whole cell lysates rather than separated mitochondrial and cytosolic fractions were utilized.

Only two caspases (caspase-3 and caspase-8) were included on the array. Interestingly, caspase-3 was not significantly changed in response to ECM treatment on the array, although blot results with individual antibodies did show a significant change in both full-length procaspase enzyme forms as well as cleaved (active) forms of the enzyme. Levels of caspase-8 were up-regulated on the antibody array in response to 1 mg/mL ECM, a response different from the results obtained using Western blotting and individual antibodies, however, functional enzyme activity assays also indicated induction of caspase-8 activity with ECM treatment.

Several members of the TNF receptor superfamily were included on the array. DR6 and Fas are both cell surface receptors belonging to the TNF receptor superfamily that were significantly up-regulated in response to ECM. Expression of DR6 was significantly increased in response to 1 mg/mL ECM, but not 2 mg/mL. DR6 is TNF receptor superfamily member 21 (TNFRSF21) and is involved in inducing apoptosis. Through its death domain, DR6 interacts with TRADD (TNFRSF1A-associated via death domain), an adaptor molecule that mediates signal transduction occurring through TNF-receptors and triggers the caspase cascade [12,22]. DR6 also activates NF-κB and MAPK8/JNK. Fas, another member of the TNF-receptor superfamily (TNFRSF6), was also significantly up-regulated in response to 1 mg/mL ECM, but not 2 mg/mL. Fas, also known as CD95 or APO-1, plays a central role in regulation of apoptosis and contains a death domain [12]. The interaction of Fas with FasL forms a death-inducing signaling complex that includes Fas-associated death domain protein (FADD), caspase-8 and caspase-10, and leads to apoptosis [12]. Results indicate activation of Fas and DR6 by ECM, thus likely leading to induction of the caspase cascade through caspase-8. Other TNF family members on the array included soluble TNF receptors sTNF-R1 and sTNF-R2, with sTNF-R2 significantly down-regulated in response to 2 mg/mL ECM. TNF-R2 mediates most effects of TNF-α [23]. TNF-α was significantly down-regulated in response to ECM treatment at 2 mg/mL. Although several TRAIL receptors, death receptors TRAIL-R1 (DR4) and TRAIL-R2 (DR5) and decoy receptors TRAIL-R3 (DcR3) and TRAIL-R4 (DcR2), were included on the array, none of these were significantly altered in response to ECM treatment. TRAIL-R1 (DR4) is activated by TRAIL and functions in transducing cell death signals and inducing apoptosis [12]. TRAIL-R2 (DR5) is also activated by TRAIL binding and transduces apoptosis signals [12]. The decoy receptor, TRAIL-R3 (DcR1), binds TRAIL but lacks a cytoplasmic death domain and therefore is not capable of inducing apoptosis. This receptor is believed to protect against TRAIL-mediated apoptosis by competing with TRAIL-R1 and R2 for ligand binding [12]. Another decoy receptor, TRAIL-R4 (DcR2), also binds TRAIL but contains a truncated cytoplasmic death domain and thus does not transmit signals for apoptosis induction [12]. Several heat shock proteins (HSP27, HSP60, HSP70), markers of cell stress, were included on the array, although none were significantly affected by ECM treatment.

Apoptosis is tightly regulated through careful balance of pro- and anti-apoptotic factors. Survivin and IGFBP-1 are apoptotic inhibitor proteins that were present on the array. IGFBP-1 has been reported to function as a negative regulator of BAK-dependent apoptosis, and thus functions to inhibit apoptosis [24]. In response to ECM treatment, IGFBP-1 was significantly down-regulated at 2 mg/mL. Based on its role as an inhibitor of apoptosis, this decrease in IGFBP-1 protein levels indicates ECM facilitates apoptosis by decreasing inhibitor expression. Although a number of pro-apoptotic molecules were positively up-regulated, a significant increase in the anti-apoptotic molecule, survivin, was observed in response to 1 mg/mL ECM. Survivin, also known as BIRC5, is an inhibitor of apoptosis and functions in promoting cell survival by inhibiting activities of caspase-3 and caspase-7 [25,26]. Survivin is a member of the IAP (inhibitor of apoptosis proteins) family of proteins and acts downstream of mitochondria to prevent processing of caspase-9 from the apoptosome, which prevents activation of downstream effector caspases, and is thought to modulate both extrinsic and intrinsic apoptotic pathways [25,26]. In contrast to malignant tissues, survivin is not usually expressed in normal cells, thus making it an ideal target for cancer therapy [27]. In experiments reported here, ECM treatment significantly increased survivin expression at 1 mg/mL, observations which are inconsistent with the large amount of data indicating activation of pro-apoptotic molecules. Despite up-regulation of survivin, however, ECM induces apoptosis in Jurkat cells. The explanation for this is unclear, although it appears that the pro-apoptotic signaling activated by ECM overcomes the anti-apoptotic signaling provided by survivin. Similar responses have been observed with another potential therapeutic agent, P2-341 (Bortezomib), in which survivin expression was increased in presence of apoptosis, an observation hypothesized to be a consequence of proteasome inhibition [28]. In other systems, Fas-induced apoptosis was associated with release of cytochrome c as well as up-regulation of survivin in mitochondria, nucleus, and cytosol [29]. Survivin is known to be a multifunctional protein with roles in cell division as well as apoptosis [30]. Survivin biology includes a link to multiple pathways of cellular homeostasis [31]. XIAP binds survivin that is released from mitochondria in response to cell death stimuli [32]. Since XIAP was dramatically decreased, it is possible that the increase in survivin was a reflection of lower amounts of available XIAP. Alternatively, the observed increase in survivin expression may be related to one of the other cellular roles attributed to survivin. Overexpression of survivin has been reported to be more efficient at blocking mitochondrial but not death-receptor-induced apoptosis [33]. Also of interest is that a complex between survivin and caspase-9 has been demonstrated [34]; survivin is also believed to associated with Smac/DIABLO [35].

### 2.6. Gene Expression

**Figure 11:**
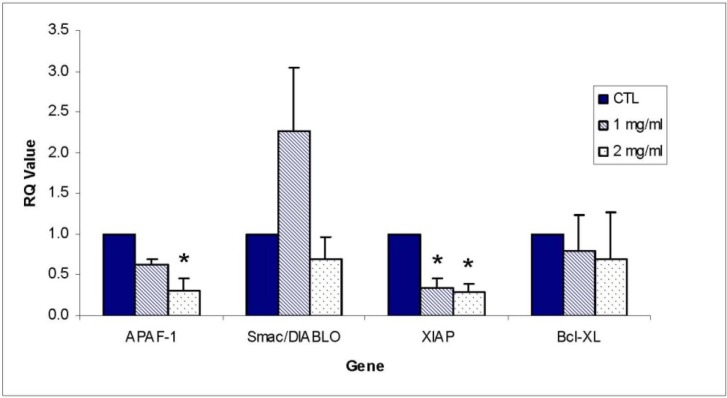
Effect of ECM treatment on gene expression on APAF-1, Smac/DIABLO, XIAP, and Bcl-xL using qPCR. * Significantly different from control (ANOVA, *P* < 0.05).

Effects of ECM treatment on the expression of APAF-1, Smac/DIABLO, XIAP, and Bcl-xL genes in Jurkat cells was investigated using both gene-specific primers and PCR arrays (described below). These results using real-time quantitative PCR are shown in Figure 11. The genes that were significantly altered included XIAP, which was significantly decreased in response to ECM treatment at 1 and 2 mg/mL and APAF-1, which was significantly decreased in response to 2 mg/mL. Genes coding for Smac/DIABLO and Bcl-xL were not significantly affected by ECM treatment. The significant decrease in gene expression for XIAP corresponds to what was observed at the protein level with the individual antibody to XIAP. The significant decrease in APAF-1 observed at the gene level, however, is contrary to what was observed at the protein level.

### 2.7. PCR Array

**Figure 12:**
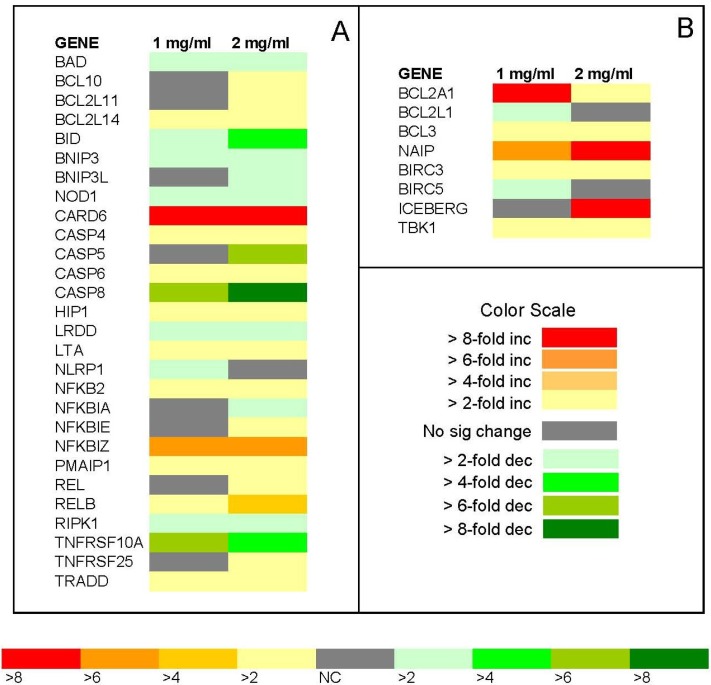
Relative fold change in expression of apoptotic pathway genes in Jurkat cells following 24 h treatment with ECM. Gene expression was determined using a PCR array (Applied Biosystems) containing primers for 84 different genes for apoptotic pathway intermediates. Results are expressed as relative fold change compared to control (untreated) Jurkat cells using a heat map according to the color scale. (**a**) relative fold change of pro-apoptotic intermediates; (**b**) relative fold change of anti-apoptotic intermediates. *N* = 3 trails for the experiment. Only genes that were significantly different among treatments (*P* < 0.05, *N* =3) are included in the heat map.

The relative fold change in expression of apoptotic pathway genes in Jurkat cells following 24 h treatment with ECM is shown in Figure 12. Gene expression was determined using a PCR array (Applied Biosystems) containing primers for 84 different genes coding for apoptotic pathway intermediates. Results are expressed as relative fold change compared to control (untreated) Jurkat cells using a heat map. Only genes with expression that was significantly changed (*P* < 0.05) in response to ECM treatment at 1 or 2 mg/mL were included on the heat map. Genes that encode positive inducers of apoptosis are shown in Figure 12A; inhibitors of apoptosis are shown in Figure 12B. The first several genes listed in the array data in Figure 12A encode mitochondrial proteins (BAD, BCL10 BCL2L11, BCL2L14, and BID). Expression of the genes encoding BAD, a mitochondrial protein that promotes apoptosis, was down-regulated in response to 1 and 2 mg/mL ECM treatment; an opposite effect to that observed at the protein level. Expression of mitochondrial BCL family members BCL10, BCL2L11, BCL2L14, all inducers of apoptosis, was up-regulated in response to 2 mg/mL ECM. BCL10 contains a caspase recruitment domain (CARD) and has been shown to induce apoptosis through activation of pro-caspase-9 and NF-κB. BCL2L11, also known as BIM, encodes a protein that acts as an apoptotic activator, as does BCL2L14. A gene coding for another mitochondrial protein and BCL2 family member, BID, was down-regulated in response to 1 and 2 mg/mL ECM. BID functions by mediating mitochondrial damage induced by caspase-8 and ultimately triggering cytochrome c release [21].

The genes BNIP3, BNIP3L, and NOD1 were all down-regulated. BNIP3 and BNIP3L are apoptosis-inducing proteins that can overcome BCL2 and BCL-XL suppression [36]. Nods, a growing family of proteins containing a nucleotide-binding oligomerization domain (NOD), are involved in regulation of programmed cell death and immune responses. APAF-1, ced-4, and Nod1 are members of this family. The NOD module is homologous to ATP-binding cassette (ABC) found in a large number of proteins with diverse biological function, and result in activation of diverse signaling pathways involved in elimination of cells via programmed cell death. NOD1 encodes a cytosolic APAF-1 like molecule that contains a caspase recruitment domain (CARD) and promotes apoptosis by enhancing caspase-9-mediated apoptosis [37]. The most strongly up-regulated molecule on the array was CARD6, with greater than 8-fold increase in expression at both 1 and 2 mg/mL ECM. The protein encoded by this gene contains a CARD with a domain structure not shared by other CARD proteins and is a microtubule-associated protein that interacts with receptor-interacting protein kinases (RIPK) and positively modulates signal transduction pathways activating NF-κB [38,39]. Most proteins containing a CARD are involved in pathways regulating apoptosis [38,39]. Examples of prominent CARD proteins are caspase-9 and APAF-1, which are involved in the intrinsic death pathway; BCL10 and CARD11, which mediate antigen receptor-induced NF-κB activation, and receptor-interacting protein (RIP)-like interacting caspase-like apoptosis regulatory protein kinase (RICK) and the nucleotide-binding oligomerization domain (NOD) proteins, which induce NF-κB activation [38].

With regard to caspase genes on the array, the genes coding for CASP4 and CASP6 were up-regulated, whereas the genes coding for CASP5 and CASP8 were down-regulated. Caspase-8 was the only caspase that appeared on both the protein and gene arrays. CASP8 was down-regulated in the gene array and up-regulated on the protein array. CASP4, CASP5, CASP6, and CASP8 all encode caspase enzymes which are initially present in the cell as inactive precursor forms that become activated with cellular processing; overexpression of these genes promotes cell death. HIP1, also known as huntingtin interacting protein, was up-regulated. The protein encoded by this gene is important in cell filament networks and promotes apoptosis through the intrinsic apoptosis pathway [40]. The gene coding for LRDD, also known as PIDD (p53-induced death domain protein), was down-regulated. LRDD is involved in apoptosis through interaction with death domain proteins such as FADD and MADD and may function as an adaptor protein in cell death-related signaling processes and play a role as an effector of p53-dependent apoptosis by promoting apoptosis as a component of the DNA damage/stress response pathway that connects p53 to apoptosis [41]. PIDD is implicated in activation of pro-caspase-2 and may mediate apoptosis induction by tumor suppressor p53 [42]. LTA encodes a protein, lymphotoxin alpha—a member of the tumor necrosis factor family, that functions as a death receptor ligand and plays a role in promoting apoptosis by binding to TNFRs [43]. LTA was up-regulated in response to both 1 and 2 mg/mL ECM. NLRP1 is a gene that encodes a member of the ced-4 family of apoptosis proteins; ced-family members contain a CARD and are known to be key mediators of programmed cell death by enhancing APAF-1 and cytochrome c-dependent activation of pro-caspase 9. NLRP1 was up-regulated in response to 1 mg/mL ECM, but unchanged in response to 2 mg/mL ECM. The higher expression of genes for certain caspase enzymes, genes coding for death domain proteins, and genes coding for proteins that promote apoptosis through death receptors in response to ECM treatment indicates apoptosis induction in target cells by ECM, proceeding through death receptor-initiated mechanisms.

The transcription factor NFκB is activated in response to death receptor signaling [44]. The pattern of NFkB pathway activation in Jurkat cells treated with ECM was complex. In the NFκB family, some mediators were up-regulated and some were down-regulated in response to ECM treatment. NFKB2, NFKBIE, and NFKBIZ were up-regulated. NFKB2, a subunit of the NFκB transcription factor complex, was up-regulated. REL (also designated c-Rel) was up-regulated, which functions in promoting cell death [44]; RELB was also up-regulated. Certain NFkB pathway regulators, NFKBIE and NFKBIZ, were also up-regulated. PMAIP, also known as NOXA, was up-regulated. This molecule is known to promote activation of caspases, and thus induction of apoptosis. PMAIP1, REL, and RELB were up-regulated. PMAIP1 promotes activation of caspases and mitochondrial membrane changes that result in efflux of apoptogenic proteins from the mitochondria, while REL and RELB form part of NFKB complex.

RIPK1, also known as receptor (TNFRSF)-interacting serine-threonine kinase, was down-regulated. The protein encoded by this gene induces apoptosis following death receptor ligation and forms a necroptosis inducing complex [45]. TNFRSF10A, also known as DR4 or TRAIL-R1, is activated by TRAIL and transduces cell death signals and induces apoptosis [43]. TNFRSF10A was down-regulated on the gene array in response to both 1 and 2 mg/mL ECM. TNFRSF25 was up-regulated in response to 1 mg/mL ECM and TRADD was up-regulated in response to both 1 and 2 mg/mL ECM. TNFRSF25, also known as DR3, regulates apoptosis by interacting directly with TRADD and activating NFκB [46]. Overexpression of TRADD (TNFR1-associated death domain protein) is involved in Fas-induced cell death pathway and activation of NFκB [46].

BCL2A1, an inhibitor of apoptosis, was up-regulated in response to both 1 and 2 mg/mL. BCL2A1 is a member of the BCL2 family and encodes a protein that decreases mitochondrial release of cytochrome c and blocks caspase activation [47]. Another apoptosis inhibitor, BCL2L1, was down-regulated. BCL2L1 also works by preventing release of caspase activators from the mitochondrial membrane and thus preventing activation of caspases [21]. Other inhibitors, BCL3, NAIP, and BIRC3, were up-regulated. NAIP (NLR family, apoptosis inhibitory protein) is involved in suppressing apoptosis by inhibiting activities of caspases-3, -7, and -9 [48]. BIRC3 encodes a member of the IAP family of proteins that inhibit apoptosis by binding to tumor necrosis receptor-associated factors TRAF1 and TRAF2 [49]. BIRC5, also known as survivin, was down-regulated on the PCR array in response to 1 mg/mL ECM, but was up-regulated at the protein level on the antibody array in response to treatment with the same concentration of ECM. ICEBERG was strongly (>8-fold) up-regulated in response to 2 mg/mL ECM. ICEBERG is a CARD family member that inhibits caspase-1 [50]. In other studies, ICEBERG was up-regulated in response to TNF [50]. TBK1 (TANK-binding kinase 1), which is also known as NF-kappa-B-activating kinase, was up-regulated; this molecule plays a role in mediate NFKB activation in response to certain growth factors.

### 2.8. Expression of TRAIL Receptors on Jurkat Cells Treated with ECM

TRAIL, tumor necrosis factor-related apoptosis-inducing ligand, is a member of the TNF family of proteins [51]. TRAIL activates apoptosis through binding to receptors known as “death receptors”. The primary known death receptors are TRAIL-R1 (also designated as DR4) and TRAIL-R2 (also known as DR5), which are members of the TNFR superfamily [52,53,54]. TRAIL also binds two other cell receptors which are known as “decoy” receptors. These decoy receptors are designated TRAIL-R3 (or DcR1) and TRAIL-R4 (or DcR2) and contain substantial homology in their extracellular domains to TRAIL-R1 and TRAIL-R2. Because of their homology, TRAIL readily binds to TRAIL-R3 and TRAIL-R4, but because they have either a truncated (DcR2) or absent (DcR1) intracellular death domain, binding of TRAIL to these receptors does not trigger apoptosis [12]. Unlike TRAIL-R1 and TRAIL-R2, neither TRAIL-R3 nor TRAIL-R4 contain intact cytoplasmic death domains that signal for apoptosis. TRAIL-R4 possesses a partially truncated death domain, whereas TRAIL-R3 is floated on the membrane via glycosyl-phosphatidylinositol linkage [55]. In cells that are sensitive to TRAIL-induced apoptosis, increased expression of decoy receptors on normal cells is believed to be a major factor responsible for resistance against TRAIL-induced apoptosis [44,56].

**Figure 13:**
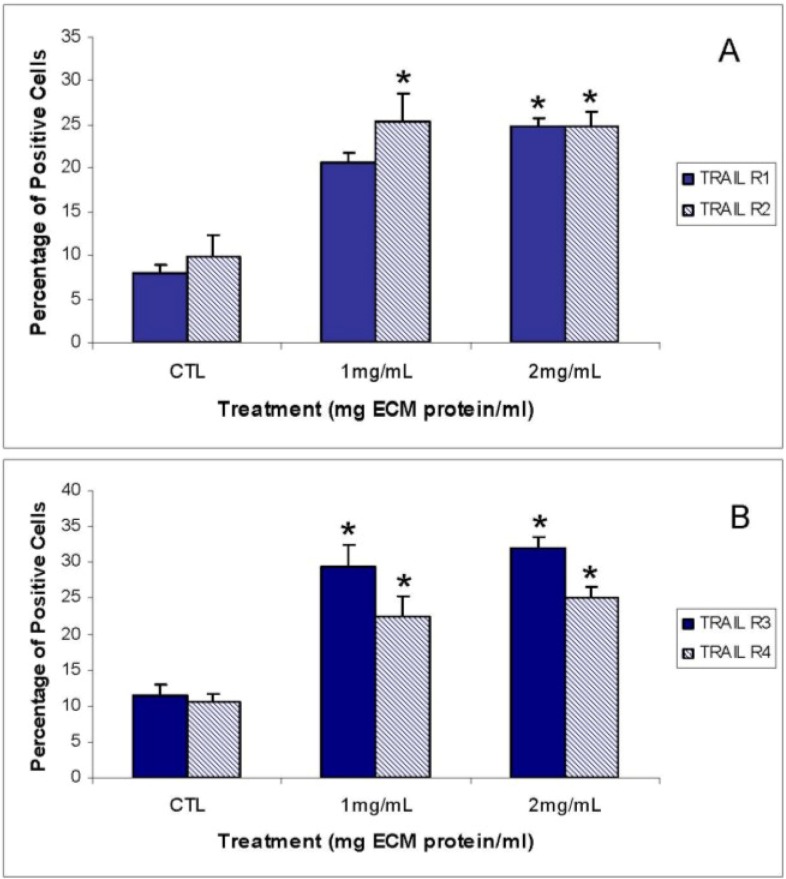
Expression of death and decoy receptors on the surface of untreated Jurkat T cell leukemia cells (control) and Jurkat cells treated with ECM at 1 and 2 mg/mL for 24 h. (**a**) death receptor (TRAIL-R1 and TRAIL-R2) expression; (**b**) decoy receptor (TRAIL-R3 and TRAIL-R4) expression. * Significantly different (*P* < 0.05; one-way ANOVA; *N* = 3) from control.

In these experiments, expression of two death receptors, TRAIL-R1 and TRAIL-R2, and two decoy receptors, TRAIL-R3 (DcR1) and TRAIL-R4 (DcR2) were examined. Jurkat cells are known to express high levels of TRAIL-R2 [57]. Expression of TRAIL-R1 (DR4) and/or TRAIL-R2 (DR5) was up-regulated in cancer cells in response to a number of chemotherapeutic drugs [58]. Surface expression of death receptors TRAIL-R1 (DR4) and TRAIL-R2 (DR5) on Jurkat cells in response to ECM is shown in Figure 13. Expression of TRAIL-R2 (DR5) was significantly (*P* < 0.05) up-regulated in response to 1 and 2 mg/mL ECM (25.35% ± 3.26%; 24.75% ± 1.65%) whereas expression of TRAIL-R1 (DR4) was significantly up-regulated only in response to 2 mg/mL ECM (24.84% ± 0.98% SEM). Surprisingly, expression of TRAIL-R3 (DcR1) and TRAIL-R4 (DcR2) were significantly up-regulated in response to both 1 and 2 mg/mL ECM.

### 2.9. Comparison of Apoptosis Pathway Intermediates Using Different Means of Detection

**Figure 14:**
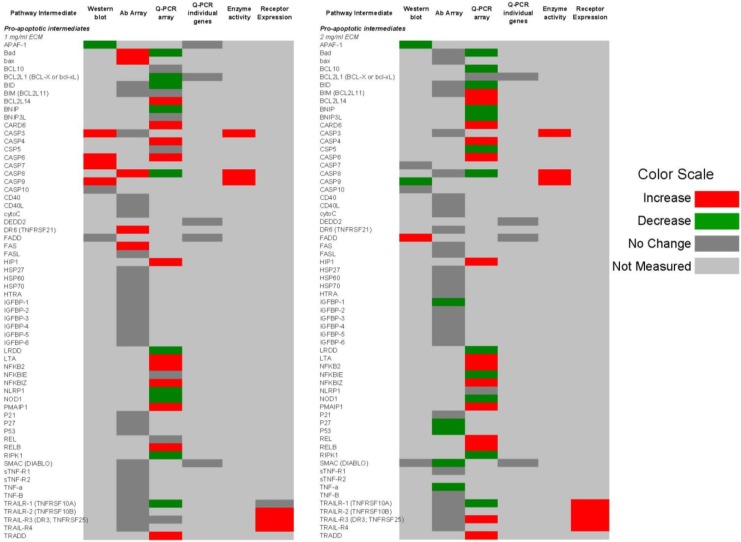
Qualitative comparison of changes in 62 pro-apoptotic regulators in Jurkat T cell leukemia cells exposed to ECM at 1 and 2 mg/mL. Observed increases or decreases are based on antibody recognition of pathway intermediates using Western blotting with individual antibodies and antibody arrays, gene expression of pathway intermediates using Q-PCR of individual genes and PCR arrays, functional enzyme activity assays, and surface expression of receptors using flow cytometric detection.

**Figure 15:**
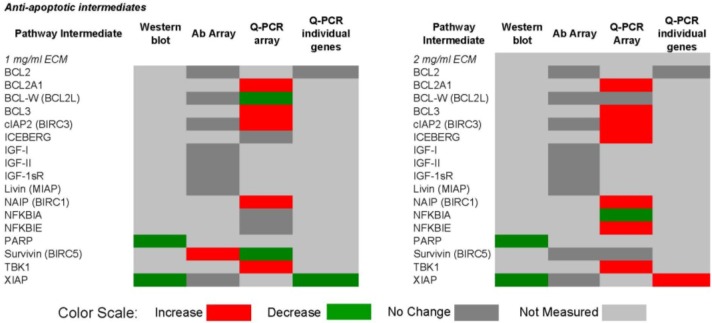
Qualitative comparison of changes in 17 anti-apoptotic regulators in Jurkat T cell leukemia cells exposed to ECM at 1 and 2 mg/mL. Observed increases or decreases are based on antibody recognition of pathway intermediates using Western blotting with individual antibodies and antibody arrays, and gene expression of pathway intermediates using Q-PCR of individual genes and PCR array.

Figure 14 and Figure 15 show qualitative comparisons of effects of 1 and 2 mg/mL ECM on apoptosis mediators in Jurkat cells as measured by different methods. Observed increases or decreases of pro-apoptosis regulators (Figure 14) and anti-apoptosis regulators (Figure 15) among Western blotting with individual antibodies and antibody arrays, gene expression of pathway intermediates using Q-PCR of individual genes and PCR arrays, functional enzyme activity assays, and surface expression of receptors using flow cytometric detection are shown.

In these comparisons, inconsistencies among results obtained through different methods of analysis were observed. Some variations may be a result of the temporal nature of apoptotic signaling, with gene expression changes occurring generally earlier than changes reflected at the protein level.

With regard to mitochondrial proteins, BAD was up-regulated in response to 1 mg/mL at the protein level but down-regulated at the gene level in response to both 1 and 2 mg/mL treatments. BID showed no change in response to 1 mg/mL at the protein level, but was decreased in expression at the gene level. In response to 2 mg/mL, BIM was decreased at the protein level but increased at the gene level. BAX was up-regulated at the gene level. BAX and BAD are important pro-apoptotic in the mitochondria [59].

With caspase enzymes, expression of caspase-8 was increased at the protein level and at the level of functional enzyme activity. Genetic expression of caspase-8, however, was decreased in response to 1 mg/mL. With 2 mg/mL ECM treatment, caspase-8 expression was decreased at both protein and gene levels. Caspase-3 expression was not significantly changed at the protein level as determined using the antibody array, but functional enzyme activity and amount of cleaved caspase-3 at the protein level were increased in response to 2 mg/mL ECM.

Expression of TRAIL-R1 was not changed at the protein level, but was decreased at the gene level. Surface expression was also not significantly up-regulated in response to 1 mg/mL ECM. Jurkat cells express higher levels of TRAIL-R2 than TRAIL-R1 [56,57]. Surface expression of TRAIL-R1 and TRAIL-R2, however, was significantly increased with 2 mg/mL ECM. No changed was observed at the protein level in expression of the decoy receptor TRAIL-R3, but an increase in expression was observed at the gene level in response to 2 mg/mL. Surprisingly, surface expression of the decoy receptors TRAIL-R3 and TRAIL-R4 was significantly increased. The observed effects with decoy and death receptors are difficult to interpret. In other studies, decoy receptors DcR1 and DcR2 were expressed on Jurkat cells and were increased in expression with stimulation with TRAIL [60]. Generally, it has been believed that transient overexpression of DcR1 or DcR2 in TRAIL-sensitive tumor cells prevents cell death trigger by TRAIL [61,62] and recent evidence indicates that tumor and normal cells can acquire resistance to TRAIL-induced killing by up-regulating TRAIL antagonistic receptors [55,63,64]. It has been proposed that decoy receptors actually are likely to play more of a regulatory rather than inhibitory role [55].

**Figure 16:**
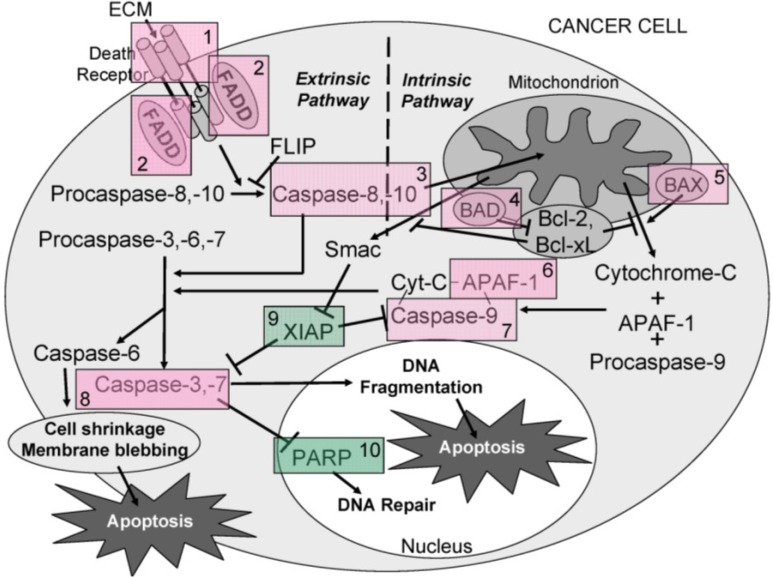
A schematic diagram showing the proposed ECM-induced regulation of pathway intermediates resulting in apoptosis in ECM-treated Jurkat cells. In this proposed schematic, ECM binds to death receptors DR4 and DR5 on the cell surface (**Box 1**) which stimulates the adapter molecule FADD (**Box 2**) to activate initiator caspases -8 and -10 (**Box 3**), mitochondrial proteins BAD (**Box 4**) and BAX (**Box 5**), post-mitochondrial apoptosis pathway intermediate APAF-1 (**Box 6**) and initiator caspase-9 (**Box 7**), resulting in the activation of terminal effector caspase-3 (**Box 8**). These data are consistent with progression of apoptosis through the intrinsic (mitochondrial) pathway. Down-regulation of an apoptotic inhibitor XIAP (**Box 9**) and cleavage of the nuclear DNA repair enzyme PARP (**Box 10**) also facilitate progression of apoptosis.

Overall, the results obtained indicate that ECM is cytotoxic (MTT) towards cultured Jurkat cells through triggering apoptosis (annexin V). The diagram in Figure 16 can be used to illustrate proposed ECM-induced regulation of pathway intermediates resulting in apoptosis in ECM-treated Jurkat cells. Briefly, results presented here following ECM treatment are consistent with apoptotic activation pathways that would be observed with binding to death receptors (DR4 and DR5) on the cell surface (Figure 16, Box 1). Based on the pattern of adaptor molecule (FADD; Box 2) and initiator caspase activation (caspase 8 and caspase 10; Box 3), activation of mitochondrial proteins BAD (Box 4) and BAX (Box 5), post-mitochondrial apoptosis pathway intermediates APAF-1 (Box 6) and caspase-9 (Box 7), and activation of the terminal caspase, caspase-3 (Box 9), these data are consistent with progression of apoptosis through the intrinsic (mitochondrial) pathway. Down-regulation of an important inhibitor of apoptosis (XIAP, Box 8) and cleavage of the nuclear DNA repair enzyme, PARP (Box 10) also facilitate progression of apoptosis.

## 3. Experimental Section

### 3.1. Animals

Mature bonnethead sharks (*Sphyrna tiburo*) were collected in the Gulf of Mexico in nearshore waters off Sarasota, FL, under a Special Activities License issued to Mote Marine Laboratory by the Florida Fish and Wildlife Conservation Commission, and transported live to Mote Marine Laboratory, in Sarasota, FL, USA.

### 3.2. Culture of Elasmobranch Epigonal Cells

Epigonal organs were excised under aseptic conditions from sharks euthanized using methods approved by the AVMA Guidelines for the Euthanasia of Animals (2013 Edition), and following protocols approved by Mote Marine Laboratory’s IACUC. Epigonal tissue was rinsed thoroughly with sterile elasmobranch-modified phosphate buffered saline (E-PBS) [65], epigonal cells were resuspended in sterile elasmobranch-modified RPMI (E-RPMI) [5,6], and cultured at 25 °C, 5% CO_2_ for 2–4 days as described [6].

### 3.3. Preparation of Epigonal Conditioned Medium (ECM)

After 2–4 days of culture, cell-free-ECM was prepared by aspirating culture supernatants and centrifuging at 2000 × *g* for 20 min at 4 °C. Cell pellets were discarded and supernatant dialyzed against several changes of 50 mM ammonium bicarbonate at 4 °C for 4–5 days using 6–8000 MWCO dialysis tubing (Spectrum). Dialyzed conditioned media preparations were lyophilized at stored at −80 °C. Protein concentration of ECM was determined using the Bradford assay (BioRad).

### 3.4. Cell line

Jurkat cells, clone E6-1, were obtained from the American Type Culture Collection (TIB-15; ATCC, Manasass, VA, USA) and maintained in RPMI-1640 (ATCC) with 10% heat-inactivated fetal bovine serum (HI-FBS; Hyclone) in a humidified atmosphere of 37 °C, 5% CO_2_. Cells were subcultured by adjusting cell concentration to 2 × 10^5^ cells/mL every 2–3 days.

### 3.5. Growth Inhibition Assay

Growth inhibitory activity of ECM against Jurkat cells was assessed using 1-(4,5-dimethylthiazol-2-yl)-3,5-diphenylformazan (MTT) [9]. Lyophilized ECM samples (*N* = 11) were resuspended in RPMI 1640 at a concentration of 4 mg/mL and filter-sterilized through a 0.2 µm syringe filter. Cells (2.5 × 10^4^ cells/mL final concentration) were co-cultured with ECM in 96-well sterile microtiter plates in triplicate for concentrations ranging from 0.25 to 2 mg/mL protein in a total volume of 200 µL RPMI 1640 and 10% HI-FBS for 72 h at 37 °C, 5% CO_2_ in a humidified atmosphere. After 72 h incubation, 100 µL supernatant was removed from each well, and microtiter plates were returned to the incubator for 4 h. After 4 h, 100 µL solubilizer (0.01 N HCl, 10% SDS) was added to each well and plates incubated overnight at 37 °C. MTT conversion was measured at 570 nm with background absorbance at 630 nm using a microplate reader (BioTek, Model ELx800, Winooski, VT, USA). Percent growth inhibition was calculated using the following formula: %GI = (CTL Abs_570-630_ − TRT Abs_570-630_ × 100).

### 3.6. Experimental ECM Exposures

Jurkat cells were co-cultured with ECM for 24 h and harvested as appropriate for analysis by enzyme activity assay, western blotting, antibody array, Q-PCR, PCR array, or cell flow cytometry. Jurkat cells were plated in sterile 24-well plates at concentrations of either 2 × 10^6^ cells/well (Q-PCR), 5 × 10^6^ cells/well (enzyme activity, Western blotting, antibody array, cell flow cytometry) or as specified below. Lyophilyzed ECM samples were resuspended in RPMI 1640 at a concentration of 4 mg/mL and filter-sterilized through a 0.2 µm syringe filter. Cells were co-cultured with ECM in concentrations of 0, 1, and 2 mg/mL protein in a total volume of 1 mL RPMI 1640 and 10% HI-FBS. Cells were incubated for 24 h at 37 °C, 5% CO_2_ in a humidified atmosphere.

### 3.7. Annexin V

Induction of apoptosis in Jurkat cells was measured using the ApoDETECT Annexin V-FITC kit (Life Technologies, Grand Island, NY, USA) according to manufacturer’s instructions. Jurkat cells were cultured as described above for flow cytometry and exposed to three different ECM (0, 1, 2 mg/mL protein) preparations or staurosporine (1 µM) in duplicate. A dead cell control was prepared by freezing cells (−80 °C) for 30 min, followed by heating (95 °C) for 30 min. Cells were incubated with ECM for 24 h at 37 °C, 5% CO_2_ in RPMI 1640 containing 10% FBS. After treatment, cells were harvested by centrifugation, washed in sterile PBS, resuspended in binding buffer, and stained with FITC-labeled Annexin V (10 µL) and propidium iodide (1 µg/mL final concentration). Samples were analyzed using an Accuri C6 Flow Cytometer with CFlow PLUS software (BD Biosciences, San Jose, CA, USA), with 25,000 events collected on each sample. Compensation was adjusted to minimize overlap in fluorescence signals. Percent apoptosis was calculated as the percentage of the total cell population which stained positive for Annexin V, but not propidium iodide.

### 3.8. Caspase Activity

Lysates of Jurkat cells were prepared using lysis buffers specific for each activity assay provided with enzyme activity kits. Protein in cell lysates was determined using the Lowry method (BioRad DC) [66]. Activity of caspase-3, caspase-8, and caspase-9 was measured in lysates of Jurkat cells treated with ECM for 24 h as described above using fluorimetric (caspase-3) and colorimetric (caspase-8 and -9) microtiter plate based assays.

#### 3.8.1. Caspase-3

Activity of caspase-3 was measured using a fluorescent microtiter plate assay (Invitrogen) based on cleavage of a non-fluorescent substrate, rhodamine 110 bis-(*N*-CBZ-l-aspartyl-l-glutamyl-l-valyl-l-aspartic acid amid, Z-DEVD-R110) by active caspase-3. With enzymatic cleavage by caspase-3, the nonfluorescent bisamide substrate is converted to fluorescent R110. Jurkat cells (20,000 per well) were treated with 0, 1, and 2 mg/mL ECM for 3 and 24 h. Excitation and emission were measured at 496 nm and 520 nm, respectively. Fluorescence was measured using black microtiter plates and a fluorescence microtiter plate reader (BioTek, Flx800, Winooski, VT, USA).

#### 3.8.2. Caspase-8

Caspase-8 activity was measured using a colorimetric microtiter plate based assay (Sigma). This assay, based on hydrolysis of the peptide substrate acetyl-Ile-Glu-Thr-Asp *p*-nitroaniline (Ac-IETD-pNA) by caspase-8, results in the release of p-nitroaniline (pNA) which is measured spectrophotometrically (405 nm, BioTek Model ELx800). Ac-IETD-pNA (caspase-8 substrate) was added at a final concentration of 2 mM. Initial absorbance was measured at 405 nm (T = 0) and then read at 5 min intervals for 90 min.

#### 3.8.3. Caspase-9

Caspase-9 activity was measured using a colorimetric microtiter plate based assay (R&D Systems, Inc., Minneapolis, MN, USA), with release of pNA from the caspase-9 substrate, LEHD-pNA, measured spectrophotometrically (405 nm, BioTek Model ELx800). Lysates were incubated with substrate for 1, 1.5, and 2 h at 37 °C.

### 3.9. Western Blotting

For Western blotting, lysates of Jurkat cells were prepared using Western lysis buffer (1% Triton X-100, 50 mM Tris-HCl, pH 8.0, 150 mM NaCl, 10% glycerol, 0.1 mM EDTA, 10 mM NaF, 1 mM sodium orthovanadate) with protease inhibitors (Sigma Protease Cocktail, 1 mM PMSF) added. Cells were lysed on ice, and spun at 12,500 × *g* for 15 min to remove cell debris. Supernatants were aspirated and supernatants stored at −80 °C until analysis. For enzyme activity assays, lysates of Jurkat cells were prepared using lysis buffers specific for each activity assay and provided with the enzyme activity kits. Protein in cell lysates was determined using the Lowry method (BioRad) [66]. Cell lysates from Jurkat cells exposed to ECM were loaded onto SDS-PAGE gels (12%) at a concentration of 40 µg/lane. Proteins were separated at 200 V for approximately 1 h. Proteins were transferred from gels onto PVDF membranes (BioRad) at 70 V for 90 min via wet transfer. Blots were blocked for approximately 1 h in blocking buffer (10% non-fat dry milk, NFDM) and incubated overnight in primary antibody dilution buffer (1:1000) to prevent non-specific binding. Primary antibodies and their sources are listed in Table 1. Anti-rabbit IgG HRP-linked secondary antibody (1:2000; Santa Cruz) in 1% blocking buffer (3% NFDM, 2% BSA in TBS-T) was used with 1 µL Precision Protein™ Strep Tactin-HRP conjugate (BioRad) added for imaging of standards. Imaging of blots was accomplished using enhanced chemiluminescence (ECL) with the Immun-Star Western C Kit (BioRad). As a protein loading control, blots were stripped with stripping buffer (15 g glycine, 1 g SDS, 10 mL Tween 20, pH 2.2 in 1 L), and then re-probed with HRP-β-actin (Santa Cruz) using 1:2000 dilution (in 1% blocking buffer) for overnight incubation (4 °C). Relative band densities on western blots were measured using ChemiDoc™ XRS^+^ with Image Lab™ image acquisition and analysis software (BioRad). Relative band densities of each protein sample were normalized to relative band density of β-actin within each lane. β-actin has been shown not to undergo cleavage in Jurkat cells undergoing apoptosis [67].

**Table 1:** Primary antibodies used in Western blotting in lysates from Jurkat cells treated with ECM.

Antibody	Source
Caspase-3	Cell Signaling
Caspase-6	Cell Signaling
Caspase-7	Cell Signaling
Caspase-8	Santa Cruz
Caspase-9	Cell Signaling
Caspase-10	Cell Signaling
PARP	Cell Signaling
APAF-1	Cell Signal
FADD	Abcam
FLIP	Abcam
XIAP	Cell Signaling
Smac/DIABLO	R & D Systems

### 3.10. Antibody Arrays

Antibody arrays (RayBio Human Apoptosis Antibody Array) containing 43 apoptotic pathway intermediates were used to measure effects of ECM exposure on Jurkat cells. Following experimental treatments with ECM for 24 h, cells from duplicate wells (10 × 10^6^ cells/treatment) were solubilized in lysis buffer containing protease inhibitors at 2 × 10^7^ cells/mL for 30 min with rocking at 4 °C. Cells were pelleted at 14,000 × *g* for 15 min, and supernatants aspirated. Protein concentration in supernatants was determined using the Lowry method (BioRad) [66]. Protein load per membrane was approximately 600 µg/mL total protein diluted 5-fold with blocking buffer. Membranes were blocked with blocking buffer for 30 min at room temperature. Diluted sample was applied to membranes and incubated overnight with gentle rocking at 4 °C. Membranes were washed, and a biotin-conjugated secondary antibody added. HRP-conjugated streptavidin was added for detection through enhanced chemiluminescence (ECL). Blots were imaged using ChemiDoc XRS+ gel imager (BioRad). Relative densities of antibodies were determined by comparing to protein loading control antibodies.

### 3.11. Real-Time PCR (Q-PCR) and PCR Arrays

ECM-treated Jurkat cells and untreated controls were screened in triplicate for expression of 96 different genes related to cell death and cell survival using “Human Apoptosis” PCR arrays (Applied Biosystems, Carlsbad, CA, USA). RNA was isolated from ECM-treated Jurkat cells with TRIzol (Invitrogen, Grand Island, NY, USA) according to the manufacturer’s instructions. Concentration and purity of RNA was assessed using a NanoDrop spectrophotometer (ThermoScientific, Waltham, MA, USA) and cDNA was prepared with the High Capacity cDNA Reverse Transcription kit (Applied Biosystems). Prepared cDNA was diluted with nuclease-free water and added to PCR arrays at a concentration of 25 ng/well with Taqman Gene Expression Master Mix (Applied Biosystems). Arrays were amplified using a 7500 Real time PCR system (Applied Biosystems) with the following conditions: initial denaturation step at 50 °C, 2 min and 95 °C, 10 min, followed by 40 cycles of 95 °C, 15 s, 60 °C, 1 min.

Expression of select genes was also analyzed in ECM-treated Jurkat cells and untreated control cells using gene-specific primers (Table 2). RNA was isolated as described using TRIzol (Invitrogen) and concentration and purity were determined using a NanoDrop spectrophotometer (ThermoScientific). RNA was reverse transcribed into cDNA using Superscript III First Strand Reverse Transcription kit (Invitrogen). Amplification of individual genes was conducted using specific primer sequences (Table 2). Primers for 18S, β-actin, beta-2 microglobulin (B2M), Fas, FADD, APAF-1, XIAP, Smac/DIABLO, and Bcl-xL were purchased from Integrated DNA Technologies (Coralville, IA). Copy DNA (cDNA) products were amplified by qPCR in quadruplicate wells containing 10 ng cDNA, 200 nM of each primer (combined forward and reverse sequences) and Power SYBR Green PCR Master Mix (Applied Biosystems) with a 7500 Real-Time PCR system (Applied Biosystems). Conditions for qPCR were as follows: initial denaturation step at 95 °C, 10 min, followed by 40 cycles of 95 °C, 15 s, 60 °C, 1 min. Q-PCR data (mean C_T_ values) for target genes was normalized to the average expression levels of three internal reference genes (18S, β-actin, and B2M) as previously recommended [68].

**Table 2:** Primer sequences used for real-time PCR amplification of individual genes in ECM-treated Jurkat cells.

Gene	Direction	5′-3′ sequence	Reference
18S	FWD	GCC AGG TCC TAG CCA ATG G	Applied Biosystems
	RVS	TCA GTC GCT CCA GGT CTT CA	Applied Biosystems
β-actin	FWD	TGC CGA CAG GAT GCA GAA G	
	RVS	CTC AGG AGG AGC AAT GAT CTT GA	
B2M	FWD	TGC TGT CTC CAT GTT TGA TGT ATC T	Vandesompele *et al*, 2002 [68]
	RVS	TCT CTG CTC CCC ACC TCT AAG T	
XIAP	FWD	5′ GCA CGA GCA GGG TTT CTT TAT ACT GGT G 3′	Espinosa *et al.*, 2006 [69]
	RVS	5′ CTT CTT CAC AAT ACA TGG CAG GGT TCC TC 3′	
Smac/DIABLO	FWD	5′ CCT GTG TGC GGT TCC TAT TGC 3′	Dai *et al.*, 2010 [70]
	RVS	5′ TGA TTC CTG GCG GTT ATA GAG 3′	
APAF-1	FWD	5′ CAC GTT CAA AGG TGG CTG AT 3′	Robles *et al.*, 2001 [71]
	RVS	5′ TGG TCA ACT GCA AGG ACC AT 3′	
Bcl-xl	FWD	5′ GTA AAC TGG GGT CGC ATT GT 3′	Will *et al.*, 2010 [72]
	RVS	5′ TGC TGC ATT GTT CCC ATA GA 3′	
CD95/Fas	FWD	5′-GAC CCA GAA TAC CAA GTG CAG ATG TA-3′	Petak *et al.*, 2003 [73]
	RVS	5′-CTG TTT CAG GAT TTA AGG TTG GAG ATT-3′	
FLIP	FWD	5′-GTT AGG TAG CCA GTT GG-3′	Kataoka *et al.*, 1998 [74]
	RVS	5′-CCT GCC TTG CTT CAG C-3′	

### 3.12. Surface Expression of TRAIL-R1 (DR4), TRAIL-R2 (DR5), TRAIL-R3 (DcR1), and TRAIL-R4 (DcR2)

The cell surface expression of death receptors (TRAIL-R1, TRAIL-R2) and decoy receptors (TRAIL-R3, TRAIL-R4) was measured in Jurkat cells (*N* = 3) treated with 0, 1, or 2 mg/mL ECM for 24 h using flow cytometry. After 24 h, cells were harvested and stained with fluorescently labeled (PE) antibodies to TRAIL-R1 (anti-hTRAILR1, FAB347P), TRAIL-R2 (anti-hTRAIL-R2, FAB311P), TRAIL-R3 (anti-hTRAIL-R3, FAB6320P), and TRAIL-R4 (anti-hTRAIL-R4, FAB633P). Mouse IgG (PE 1C002P) was used as isotype control. All antibodies were obtained from R&D Systems. Expression of cell surface receptors was measured using an Accuri C6 Flow Cytomter with CFLow PLUS software (BD Biosciences, San Jose, CA, USA), with 25,000 events collected on each sample.

### 3.13. Statistical Analyses

#### 3.13.1. Growth Inhibition (MTT), Annexin V, and Receptor Expression Assays

Statistical analyses comparing untreated with ECM-treated cells were performed using Sigma-Stat Version 2.03. Means ± SEM are presented. Data that passed tests for both normality and equal variance were analyzed using either one-way analysis of variance (ANOVA) or Student’s *t*-test. Data that did not satisfy requirements for normality and equal variance were analyzed using either non-parametric rank sum test or one-way ANOVA on Ranks. *P*-values less than 0.05 were considered significant.

#### 3.13.2. Western Blots and Antibody Arrays

β-actin was used as a protein loading control. Blot images were normalized to β-actin. Percent change compared to untreated control was determined. Relative band densities from three to four individual blots were measured, and data reported as mean ± SEM. Antibody arrays were normalized to three averaged protein loading controls. Tests of significance were determined using either one-way ANOVA among ECM treatment concentrations or Student’s *t*-test for comparing individual concentrations to untreated control. *P* values less than 0.05 were considered significant.

#### 3.13.3. Q-PCR and PCR Arrays

Q-PCR array data were analyzed using the RT2 Profiler PCR Array Data Analysis web-based software (Version 3.5, Qiagen). Briefly, mean C_T_ values for target genes were normalized to the average expression levels of two internal reference genes (GAPDH and beta glucuronidase (GUSB)). The resulting ΔC_T_ values were averaged for replicates within the untreated and ECM-treated groups (1 mg/mL and 2 mg/mL). Mean ΔC_T_ values for ECM-treated groups were then normalized to those of the untreated control group and used to calculate the fold change in expression. Expression changes (up- or down-regulated) greater than 2-fold with *P* < 0.05 were considered significant.

Data from gene specific Q-PCR analysis (mean C_T_ values) for target gene expression of APAF-1, Smac/DIABLO, XIAP, and Bcl-xL were normalized to the average expression levels of three internal reference genes (18S, β-actin, and B2M) as previously recommended [68]. The resulting ΔC_T_ values were averaged for replicates within the untreated and ECM-treated groups (1 mg/mL and 2 mg/mL). Mean ΔC_T_ values for ECM-treated groups were then normalized to those of untreated control group and used to calculate the relative quantity (RQ). Differences in expression between untreated and ECM-treated groups were determined by one-way ANOVA, with *P* < 0.05 indicating significant differences.

## 4. Conclusions

Sharks and their skate and ray relatives (Subclass Elasmobranchii), collectively known as the elasmobranch fishes, represent a novel and untapped source of marine-derived compounds with the potential to be developed into therapeutic agents to benefit human health. Apoptosis of Jurkat T cell leukemia cells (clone E6-1, ATCC TIB-15) occurs in response to 24 h treatment with compounds present in a culture medium conditioned by shark epigonal organ cells (epigonal conditioned medium, ECM). The data presented support the conclusion that specific apoptotic pathways are targeted by ECM compounds and may have a direct role in inducing tumor cell death and/or sensitizing cancer cells to apoptosis induced through other means.
